# Highly Conductive Transparent Organic Electrodes with Multilayer Structures for Rigid and Flexible Optoelectronics

**DOI:** 10.1038/srep10569

**Published:** 2015-05-27

**Authors:** Xiaoyang Guo, Xingyuan Liu, Fengyuan Lin, Hailing Li, Yi Fan, Nan Zhang

**Affiliations:** 1 State Key Laboratory of Luminescence and Applications, Changchun Institute of Optics, Fine Mechanics and Physics, Chinese Academy of Sciences, Changchun 130033 (China)

## Abstract

Transparent electrodes are essential components for optoelectronic devices, such as touch panels, organic light-emitting diodes, and solar cells. Indium tin oxide (ITO) is widely used as transparent electrode in optoelectronic devices. ITO has high transparency and low resistance but contains expensive rare elements, and ITO-based devices have poor mechanical flexibility. Therefore, alternative transparent electrodes with excellent opto-electrical performance and mechanical flexibility will be greatly demanded. Here, organics are introduced into dielectric–metal–dielectric structures to construct the transparent electrodes on rigid and flexible substrates. We show that organic-metal-organic (OMO) electrodes have excellent opto-electrical properties (sheet resistance of below 10 Ω sq^−1^ at 85% transmission), mechanical flexibility, thermal and environmental stabilities. The OMO-based polymer photovoltaic cells show performance comparable to that of devices based on ITO electrodes. This OMO multilayer structure can therefore be used to produce transparent electrodes suitable for use in a wide range of optoelectronic devices.

The development trends of optoelectronic products such as touch panels, organic light-emitting diodes, and solar cells will be flexible, light weight and low-cost. All these devices are constructed from transparent electrodes, which must be equipped with excellent opto-electrical performance and mechanical flexibility, while also compatibility with low-cost fabrication. Traditionally, the material of choice is indium tin oxide (ITO), which is frequently used as the transparent electrode in many optoelectronic devices because of its good combination of high transparency and low resistance^1^,^2^,^3^. However, ITO is prone to certain problems, mainly related to scarcity of supplies and its brittle nature, leading to dramatic price fluctuations and poor mechanical flexibility in flexible devices^3^,^4^,^5^,^6^. Several emerging materials such as conducting polymers^7^,^8^, carbon nanotubes^9^,^10^, graphene^11^,^12^, and metallic nanowires^13^,^14^ show potential as replacements for sputtered ITO. But there are also challenges for these new materials, such as large sheet resistance in conducting polymers, long-term stability and high contact resistances in carbon nanotubes and metallic nanowires, and large-scale fabrication in graphene^3^. Therefore, developing new materials combine most desirable properties for transparent electrodes will help to satisfy the increasing demand for low-cost flexible devices.

Compared with inorganic materials, organic materials have several advantages, including low-cost processing, mechanical flexibility, and broad spectral and energy level tunabilities. In terms of fabrication, they can be solution processed at low cost, allowing large-area deposition on flexible substrates through roll-to-roll or contact printing technologies. Organics are therefore promising candidates for large-area and flexible transparent electrodes. However, organics also have drawbacks such as low carrier densities and low charge mobilities, which limit the development of organics as transparent electrodes. A well-known emerging transparent electrode based on an organic material is poly(3,4-ethylenedioxythiophene) doped with polystyrene sulfonic acid (PEDOT:PSS). At first, PEDOT:PSS has seldom been used as a single transparent electrode in organic optoelectronic devices because of its limited electrical properties (the sheet resistance of PEDOT:PSS is typically 10^4^–10^5^ Ω sq^−1^)^15^. Afterwards, the conductivity of PEDOT:PSS can be enhanced by adding a high-boiling point solvent, increasing the ratio of PEDOT^15^,^16^,^17^. Recently, the lowest sheet resistance of PEDOT:PSS that treated by H_2_SO_4_ can be achieved is around 40-50 Ω sq^−1^, together with a transmittance of about 90%^18^,^19^. However, the optoelectronic properties of PEDOT:PSS still have a gap with commercial ITO electrode (~10 Ω sq^−1^, transmittance >85%), and need to be improved to meet the requirements of optoelectronic devices.

Previous studies have shown that the transmittance of a metal layer can be enhanced by inserting a metal layer between two dielectric layers with suitable refractive indexes^20^,^21^,^22^. The transmittance and conductivity of a multilayer transparent film based on a dielectric–metal–dielectric (DMD) structure can be optimized by tuning the thicknesses of the dielectric and metal layers to achieve high transparency in the visible region, as well as high conductivity^23^,^24^,^25^. Many inorganic semiconductors have been employed as the dielectric materials in DMD transparent electrodes that exhibited competitive properties in optoelectronic devices. Since the electrical properties of DMD electrodes mainly depend on the metal layer, there is a high possibility that DMD structure with organic dielectric materials may have a much better conductivity than most conducting polymers. Based on such kind of organic DMD electrode, a favorable electrode/organic interface can be expected in polymer photovoltaic (PV) cells that having more convenient control of interface potential barrier and active layer morphology, and a better charge collection efficiency.

In this study, organics were introduced into the DMD structure to act as the dielectric layers. Figure 1a shows the materials and structure of the organic–metal–organic (OMO) multilayer electrode. OMO electrodes with the structure poly(*N*-vinylcarbazole) (PVK)/Ag/PVK (PAP) and PVK/Ag/PEDOT:PSS (PAPE) show excellent electrical and optical properties; in particular, the sheet resistance of an OMO electrode can be adjusted to below 10 Ω sq^−1^, and the maximum transmittance is over 85%. They also show well thermal, mechanical and environmental stabilities. The OMO electrodes with the PAPE structure were successfully used in polymer PV cells, and showed characteristics comparable to those of devices based on ITO electrodes, indicating the potential for applications of OMO electrodes in rigid and flexible optoelectronic devices. Importantly, other organic semiconductors can also be employed in OMO structures to produce transparent electrodes or coloured conductive films, indicating that increasing numbers of different electrodes based on organic materials with ultralow sheet resistances can be fabricated. This will greatly contribute to the transparent electrodes, especially in flexible electrodes.

## Results

**Figure 1:**
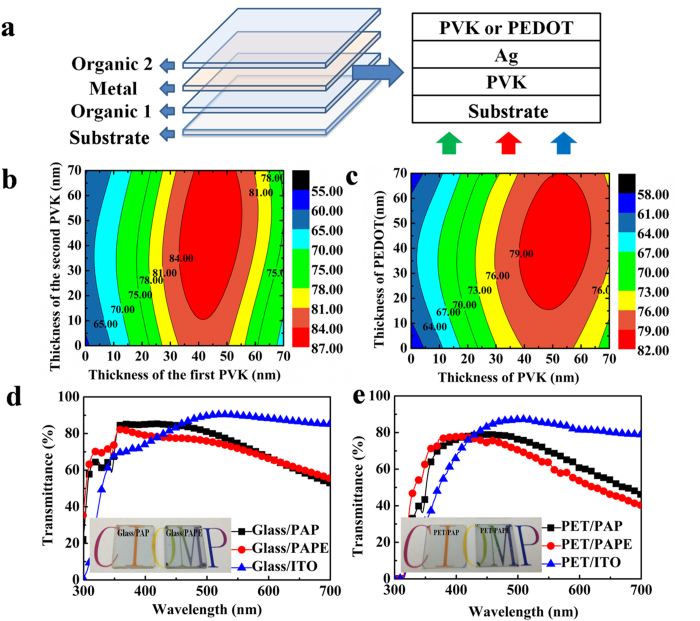
Structures and optical properties of transparent electrodes. (**a**) Structures of multilayer transparent electrodes based on organic materials. (**b**) Calculated transmittances of PVK/Ag (12 nm)/PVK and (**c**) PVK/Ag (12 nm)/PEDOT structures at 500 nm as a function of both organic layer thicknesses. Measured transmittance spectra of PVK (35 nm)/Ag (12 nm)/PVK (35 nm), PVK (35 nm)/Ag (12 nm)/PEDOT:PSS (35 nm), and ITO on (**d**) glass and (**e**) PET substrates. Photographs of PAP, and PAPE are shown in the inset of d and e.

To optimize the optical transmittances of OMO electrodes, ensuring that more photons pass through them, optical analysis was carried out using the optical transfer matrix method^26^,^27^. Figure 1b,c show the contour plots for the transmittances of OMO multilayers with the PAP and PAPE structures at a wavelength of 500 nm, as a function of PVK and PEDOT:PSS thicknesses. In this calculation, the thickness of Ag was fixed at 12 nm. The maximum calculated transmittance of PAP (Fig. 1b) on glass substrates was optimized to over 84% by varying the thicknesses of both organic layers. The best transmittance of PAP on a glass substrate was achieved at a thickness of the first PVK of 35–55 nm together with a thickness of the second PVK of over 10 nm. When the second PVK was substituted for PEDOT:PSS, the maximum calculated transmittance of PAPE (Fig. 1c) on glass substrates was reduced to over 79%. The best transmittance of PAPE on a glass substrate was achieved at a PVK thickness of 40–60 nm along with a PEDOT:PSS thickness of over 15 nm. Our calculations indicated that transparent electrodes with a DMD structure based on organics can be produced and the transmittance of the OMO structure can be enhanced to over 80% by varying the thicknesses of both organic layers.

Figure 1d,e show the transmittance spectra of PVK/Ag/PVK and PVK/Ag/PEDOT:PSS transparent electrodes on glass and PET substrates, respectively; the transmittance spectrum of ITO on glass and PET substrates are also shown for comparison. Compared with the ITO electrode, the OMO electrodes have lower transmittances at wavelengths above 450 nm, but show higher transmittances at short wavelengths of 300–450 nm; this is attributed to the wide band gap of PVK (3.4 eV)^28^. The maximum transmittances of PAP and PAPE on glass substrates are over 85% and 82%, respectively. The average transmittances of PAP and PAPE on glass substrates at 300–700 nm are 68.8% and 67.2%, respectively, which are a little lower than that of ITO (72.8%), but are higher than that of 12 nm Ag (55.2%, Fig. S1) over the same spectral range. The transmittances of PAP and PAPE on PET substrates are a little lower than those of on glass substrates, but the variation trends are similar as those of on glass substrates. The photographs of PAP and PAPE electrodes are shown in the inset of Fig.1d,e, respectively, which show that the two OMO electrodes have good transparencies on glass and plastic substrates.

**Figure 2:**
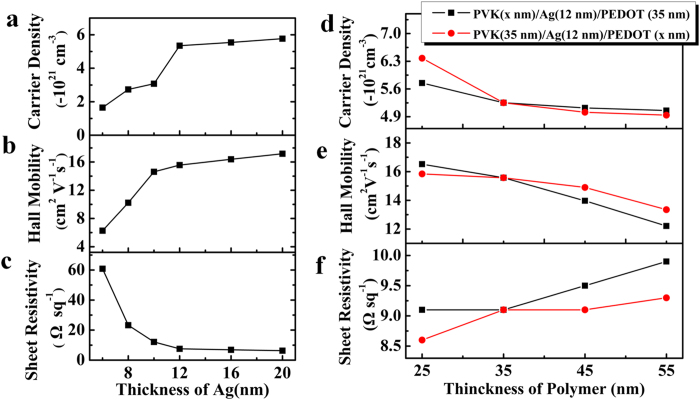
Electrical characteristics of PAPE transparent electrode. (**a**) Carrier density, (**b**) Hall mobility, and (**c**) sheet resistance of PAPE electrodes as a function of Ag thickness. The PVK and PEDOT:PSS thicknesses are both 35 nm. (**d**) Carrier density, (**e**) Hall mobility, and (**f**) sheet resistance of PAPE electrodes as a function of PVK and PEDOT:PSS thicknesses. The Ag thickness is fixed at 12 nm.

Transparent electrodes based on the OMO structure also show excellent conductivity. The effect of the thickness of each layer on the electrical properties of the PAPE structure has been studied. Figure 2 shows the electrical characteristics, namely the carrier density, Hall mobility, and sheet resistance, of the PAPE electrode as a function of each layer thickness. As can be seen in Fig. 2a–c, as the Ag thickness increases from 6 to 20 nm, both the carrier density and the Hall mobility of the PAPE electrode increase, and the sheet resistance decreases significantly from 61 to 6.3 Ω sq^−1^. The increased mobility is mainly attributed to interfacial scattering at the metal/organic interface^29^. When the Ag thickness is more than 12 nm, the sheet resistance changes little, which implies that 12 nm-thick Ag film is almost continuous. It can be seen in Fig. 2d–f that the carrier density of the PAPE electrode decreases with increasing thickness of both organic layers, and the Hall mobility of the PAPE electrode tends to increase. The sheet resistance of the PAPE electrode increases slightly with increasing thickness of the organic layer from 25 to 55 nm. These results indicate that the electrical properties of the PAPE electrode are strongly dependent on the thickness of the Ag layer. However, a thicker Ag layer decreases the transparency of the PAPE electrode. An ideal balance between the maximum transmittance and the sheet resistance is obtained when the thickness of the Ag layer is in the range 10–12 nm, achieving a maximum transmittance of over 83% and a sheet resistance of below 15 Ω sq^−1^ (Fig. S2); these values meet the requirements of most optoelectronic applications.

**Figure 3:**
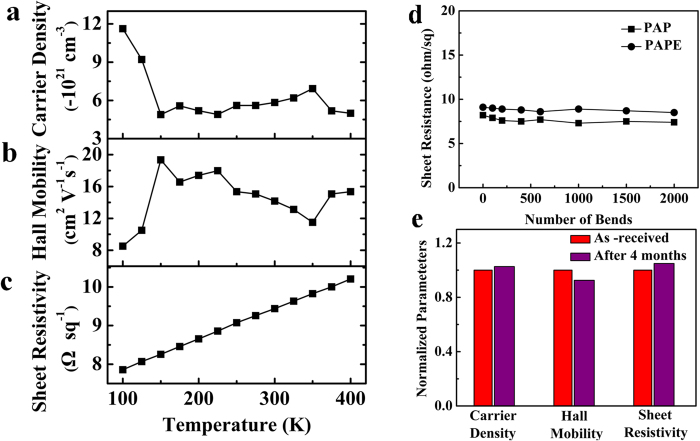
Stabilities of PAPE transparent electrode. (**a**) Carrier density, (**b**) Hall mobility, and (**c**) sheet resistance of PAPE electrode as a function of temperature. (**d**) The sheet resistivity of PET/PAP and PET/PAPE flexible electrodes under repeated bending. The bending angle is 90°. (**e**) The normalized changes of carrier density, Hall mobility and sheet resistivity of the PAP electrode before and after 4 months in air.

To investigate the effect of temperature on the electrical properties of the OMO electrode, variable-temperature Hall measurements were performed in the temperature range 100–400 K. Figure 3a-c shows the temperature dependences of the carrier density, Hall mobility, and resistivity of the PAPE electrode. The carrier density and the Hall mobility fluctuate with increasing temperature, whereas the resistivity shows a linear increase from 7.7 to 10 Ω sq^−1^ with increasing temperature. The temperature dependence of the resistivity suggests metallic behaviour of the PAPE electrode; this is mainly caused by the middle Ag layer. Anyhow, the OMO electrode PAPE exhibits stable electrical properties over a wide range of temperatures.

The mechanical and environmental stabilities of the flexible OMO electrodes have also been studied. Figure 3d shows the sheet resistances of the flexible PAP and PAPE electrodes under repeated bending. It can be seen that there is little change in sheet resistance despite 2000 bending cycles, which is clearly indicative of excellent durability of the OMO electrodes. For comparison, the same measurement was carried out with PET/ITO electrode, the sheet resistance of the flexible ITO electrode increases to about 1800 Ω sq^-1^ after only 400 bending cycles (Fig. S6). The PAP electrode also shows excellent environmental stability. The sheet resistance increases by only 5% after exposing the PAP electrode in air for four months (Fig. 3e).

**Figure 4:**
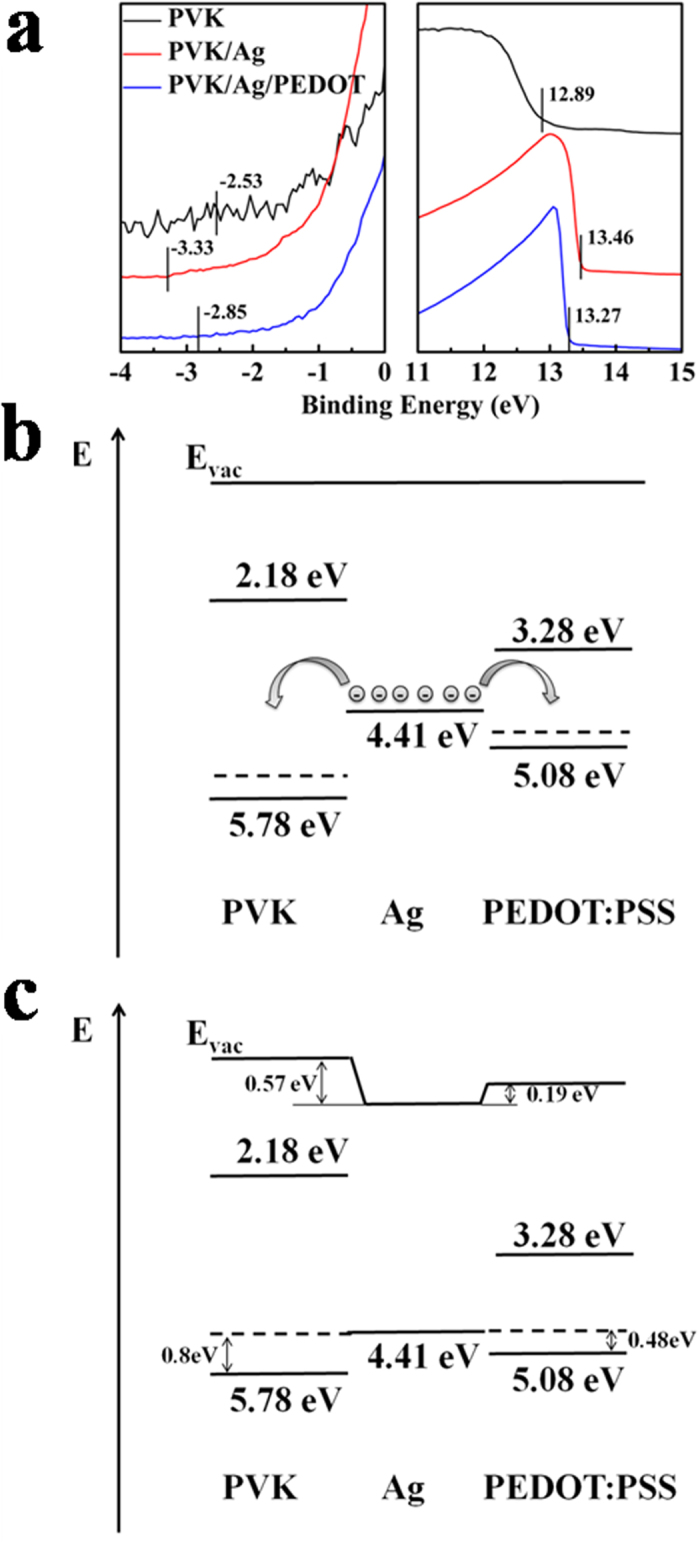
UPS and energy level alignment of PAPE electrode. (**a**) UPS of PVK, PVK/Ag and PVK/Ag/PEDOT:PSS films based on ITO substrate. Energy level alignment of PAPE electrode (**b**) before and (**c**) after contact.

In order to explore the underlying conducting physical mechanism of the multilayer structure, ultraviolet photoelectron spectroscopy (UPS) was carried out to determine the electronic structures at the interfaces between metal and organic dielectric layers. Figure 4a shows the UPS spectra of PVK, PVK/Ag and PVK/Ag/PEDOT:PSS films based on ITO substrate. And the proposed energy level alignments of the PAPE electrode before and after contact are shown in Fig. 4b,c. The values of highest occupied molecular orbital (HOMO) for PVK and PEDOT:PSS, the work function (E_F_) for Ag, and the changes in energy level for each material were extracted from the UPS spectra^30^. The values of lowest unoccupied molecular orbital (LUMO) for PVK and PEDOT:PSS were determined from UPS spectra together with their band gap calculated from their absorption spectra. As seen in Fig. 4c, after contacting, electrons at the PVK/Ag and Ag/PEDOT:PSS interface are transferred from Ag to PVK and PEDOT:PSS, respectively, resulting in a common Fermi level in the multilayer structure. However, the transferred electrons are acting only several nanometers at the metal/organic interface, doing very limited effect to the electrical characteristics of a thick organic layer. Although the conductive mechanism of the OMO electrode is not yet clear, it is confirmed that the OMO electrodes mainly conduct through the middle metal layer (Fig. S7), and further research is already underway.

**Figure 5:**
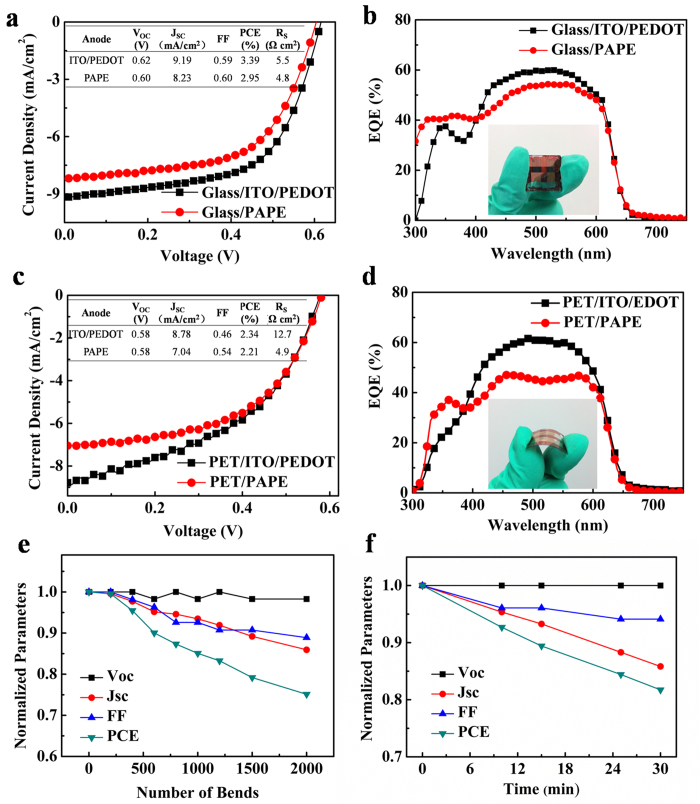
Characteristics of device based on PAPE transparent electrode. (**a**) *J*–*V* characteristics and (**b**) EQE spectra of polymer PV cells based on Glass/ITO and Glass/PAPE electrodes. (**c**) *J*–*V* characteristics and (**d**) EQE spectra of polymer PV cells based on PET/ITO and PET/PAPE electrodes. (**e**) Normalized PV parameters of a flexible polymer solar cell based on PAPE electrode as a function of bending numbers. The bending angle is 90°. (**f**) Normalized parameters of a flexible polymer solar cell based on PAPE electrode as a function of its exposure to air.

To study the properties of the OMO transparent electrode, polymer PV cells on glass and plastic substrates based on poly(3-hexylthiophene):,[6]-phenyl C_61_-butyric acid methyl ester (P3HT:PCBM) were prepared with PAPE electrodes. Figure 5a and c show the current density–voltage (*J*–*V*) characteristics of polymer PV cells based on glass and PET substrates under 100 mW cm^−2^ AM 1.5G illumination, respectively. The detailed parameters of these PV cells are summarized in the inset tables of Fig. 5a,c. For the device using Glass/PAPE as the anode, a power conversion efficiency (PCE) of 2.95% was obtained with a short-circuit current density (*J*_SC_) of 8.23 mA cm^−2^, an open-circuit voltage (*V*_OC_) of 0.60 V, and a fill factor (FF) of 0.60. For comparison, a device with an Glass/ITO/PEDOT:PSS anode was fabricated; a PCE of 3.39% was achieved, with a *J*_SC_ of 9.19 mA cm^−2^, a *V*_OC_ of 0.62 V, and a FF of 0.59. The device using PET/PAPE showed a *V*_OC_ of 0.58 V, a *J*_SC_ of 7.04 mA cm^−2^, a FF of 0.54 and a calculated PCE of 2.21%. The reference flexible cell based on PET/ITO/PEDOT:PSS anode exhibited a *V*_OC_ of 0.58 V, a *J*_SC_ of 8.78 mA cm^−2^, a FF of 0.46 and a calculated PCE of 2.34%. The series resistances (*R*_s_s) of the four devices, calculated from the *J*–*V* curves under light, are also shown in the table. Compared with the ITO/PEDOT devices (5.5 Ω cm^2^ for glass substrate and 12.7 Ω cm^2^ for PET substrate), the devices based on the PAPE anodes show lower *R*_s_s, 4.8 Ω cm^2^ for glass substrate and 4.9 Ω cm^2^ for PET substrate, respectively, resulting in higher FFs for the PAPE devices. The low *R*_s_s of the PAPE devices may originate from the low sheet resistances of the PAPE electrodes. The lower PCEs of the device with the PAPE electrodes were attributed to the smaller *J*_SC_, which was a result of the lower transmittance of the PAPE electrode at wavelengths 400–700 nm; this was confirmed by the external quantum efficiency (EQE) spectra of devices based on different electrodes (Fig. 5b,d). However, a higher EQE was obtained over the range 300−400 nm, which compensated for the loss of *J*_SC_.

The characteristics of the flexible polymer PV cell under repeated bending and exposing in air were also investigated (Fig. 5e,f). About 75% of its initial PCE was achieved after 2000 bending cycles, and about 82% of its initial PCE was achieved after exposing in air for 30 min. As the similar trends of device degradation were found in the above measurements, it can be deduced that the variation in performance under repeated bending is a normal degeneration process in air. The device performance will be enhanced through encapsulating. However, the successful use of the PAPE electrode in polymer solar cells shows that transparent electrodes based on OMO structures have potential applications in other rigid and flexible optoelectronic devices.

To further investigate whether the OMO electrodes have universal properties, other organic semiconductors such as copper phthalocyanine (CuPc) and P3HT were introduced into the OMO structure, and Au was used as the middle metal. Table 1 shows the electrical parameters of OMO electrodes with different organics and metals. It can be seen that the OMO electrodes with Ag have better properties than the OMO electrodes with Au; this is attributed to the higher conductivity of Ag and the lower thickness of Au. However, most organic semiconductors can be used in the OMO structure to obtain transparent electrodes or coloured conductive films (Fig. S8).

In summary, transparent electrodes based on organics, with the OMO structure, have been developed. An OMO transparent electrode with PAP showed excellent optical properties, with a maximum transmittance of over 85% on the glass substrate. The OMO electrode also shows excellent thermal, mechanical and environmental stabilities. The detailed resistance tests reveal that the OMO electrode is conductive on both of the horizontal and the vertical directions, which mainly depends on the middle metal layer. By varying the thickness of the organic or metal layer, the sheet resistance of the PAPE electrode can be adjusted to below 10 Ω sq^−1^, and the maximum transmittance is over 82% on the glass substrate. The PCE of polymer PV cells with Glass/PAPE and PET/PAPE as the anodes were 2.95% and 2.21%, respectively, which were comparable to those of the reference cells (3.39% for Glass/ITO and 2.34% for PET/ITO), indicating the potential for application of OMO electrodes in rigid and flexible optoelectronic devices. The most important implication of this work is that transparent electrodes based on the OMO structure can be extended to many organic semiconductors, achieving high transmittance combined with ultralow sheet resistance. This enables the fabrication of excellent transparent electrodes based on organics for low-cost and flexible optoelectronic devices.

## Methods

For the PAP structure, PVK (Sigma-Aldrich, Mw = 1100000 g/mol) was spin-coated onto a substrate (glass or PET) from chlorobenzene in a glove box. Then a thin layer of Ag was deposited by e-beam thermal evaporation at a pressure of approximately 4 × 10^−4^ Pa. The film deposition rate and thickness were monitored and controlled automatically *in situ* by a thin film deposition controller (MDC-360C). The thickness error of the Ag film was about 2%. The deposition rate was about 8-10 Å/s. Finally, the substrate was moved to the glove box, a PVK layer was spin-coated on top of the Ag layer. For the PAPE transparent electrode, the first two layers were fabricated using the same process as for PAP; the top PEDOT:PSS layer was spin-coated in air from a PEDOT:PSS (Baytron P, Lot No.: HCD06P084) solution diluted with isopropyl alcohol at a volume ratio of 1:1.

Polymer solar cells were fabricated on the PAPE transparent electrodes. A blend of a conjugated polymer, P3HT (Solarmer) and PCBM (Nichem), was spin-coated onto the substrate from chlorobenzene, in a glove box, to serve as the active layer. The substrates were heated at 160 °C and 120 °C for 10 min on a hot plate in the glove box for the glass and PET substrates, respectively. Finally, LiF (1 nm) and Al (100 nm) were thermally deposited at a pressure of 4 × 10^−4^ Pa. For comparison, devices based on ITO (12 Ω·sq^−1^ for glass substrate, or 40 Ω·sq^−1^ for PET substrate)/PEDOT:PSS (Baytron P, Lot No.: HCD06P084; diluted with isopropyl alcohol, volume ratio 1:1) electrodes were fabricated using the same process. The active area of the PV cell was 0.12 cm^2^.

Optical transmittance spectra were obtained using a Shimadzu UV-3101PC spectrophotometer. The carrier concentration, resistivity, and Hall carrier mobility of the samples (18 mm×18 mm) were obtained by Hall effect measurements with an applied magnetic filed of 0.55 T and current range from 1 nA to 1 mA. UPS was carried out using a Thermo ESCALAB 250 surface analysis system equipped with a He-discharge lamp providing He–I photons of 21.22 eV. Film thicknesses were measured using an Ambios XP-1 surface profiler. The *J*–*V* characteristics of the polymer PV cells were measured using a computer-controlled Keithley 2611 source meter under AM 1.5G illumination from a calibrated solar simulator with an irradiation intensity of 100 mW·cm^−2^. EQE measurements were performed using a lock-in amplifier at a chopping frequency of 20 Hz during illumination with monochromatic light from a Xe lamp. The air exposure lifetime study was carried out in the uncontrolled air condition (24 °C, RH 30%) under AM 1.5 G illumination with an irradiation intensity of 100 mW·cm^−2^. All measurements were performed in ambient air.

## Additional Information

**How to cite this article**: Guo, X. *et al.* Highly Conductive Transparent Organic Electrodes with Multilayer Structures for Rigid and Flexible Optoelectronics. *Sci. Rep.* **5**, 10569; doi: 10.1038/srep10569 (2015).

## Supplementary Material

Supplementary Information

## Figures and Tables

**Table 1:** Electrical parameters of OMO electrodes with different organics and metals.

**Sample**	**Carrier Density (×10**^**21**^ **cm**^**-3**^)	**Hall Mobility (cm**^**2**^**V**^**-1**^**s**^**-1**^)	**Resistivity (×10**^**-5**^ **Ω cm)**	**Sheet Resistance (Ω sq**^**−1**^)
PVK/Ag/PVK	−6.2 ± 0.2	15.3 ± 0.7	6.6 ± 0.1	8.2 ± 0.1
PVK/Ag/PEDOT	−5.3 ± 0.1	15.7 ± 0.7	7.5 ± 0.2	9.1 ± 0.2
PEDOT/Ag/PEDOT	−5.8 ± 0.1	15.0 ± 0.5	7.2 ± 0.1	9.0 ± 0.1
CuPc/Au/CuPc	−9.8 ± 0.2	4.1 ± 0.2	15.6 ± 0.3	31.2. ± 0.6
P3HT/Au/P3HT	−5.0 ± 0.2	2.6 ± 0.2	47.7 ± 0.5	68.1 ± 0.7
PVK/Au/PVK	−3.9 ± 0.1	4.7 ± 0.2	34.2 ± 0.2	42.8 ± 0.3
