# Supplementary material for: Highly Conductive Transparent Organic Electrodes with Multilayer Structures for Rigid and Flexible Optoelectronics
Source: Sci Rep. 2015 May 27;5:10569. doi: 10.1038/srep10569 (PMC4444971; doi:10.1038/srep10569)
Supplement: Supplementary Information [file srep10569-s1.pdf]

Supplementary Information

Highly conductive transparent organic electrodes with multilayer structures for rigid and flexible optoelectronics

Xiaoyang Guo, Xingyuan Liu*, Fengyuan Lin, Hailing Li, Yi Fan*, Nan Zhang

State Key Laboratory of Luminescence and Applications, Changchun Institute of Optics, Fine Mechanics and Physics, Chinese Academy of Sciences, Changchun 130033 (China)

Corresponding Author

*E-mail: liuxy@ciomp.ac.cn; fanyi@ciomp.ac.cn

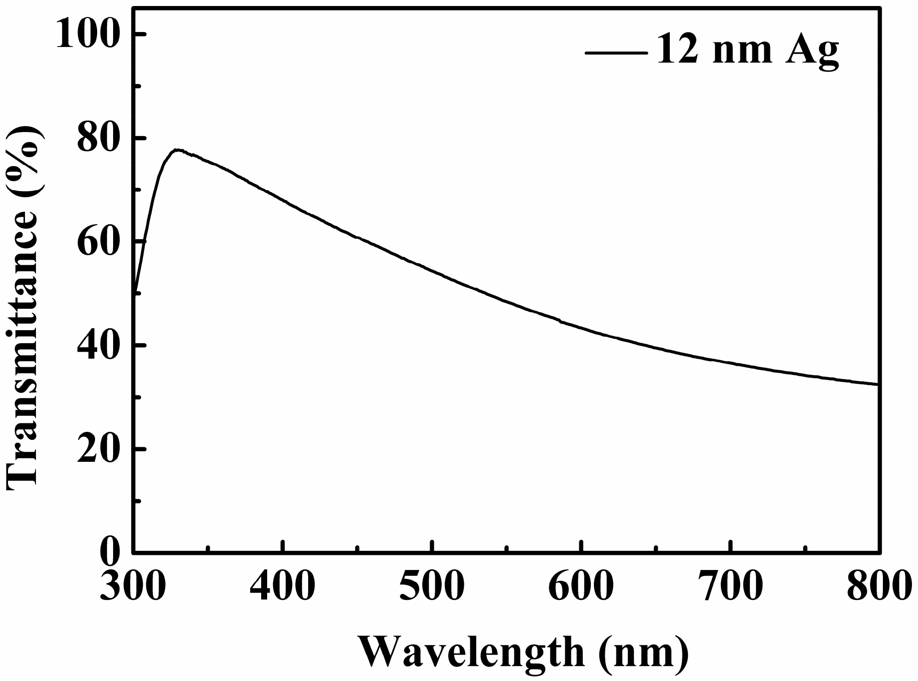

Figure S1. Transmittance of 12 nm-thick Ag film on glass substrates.

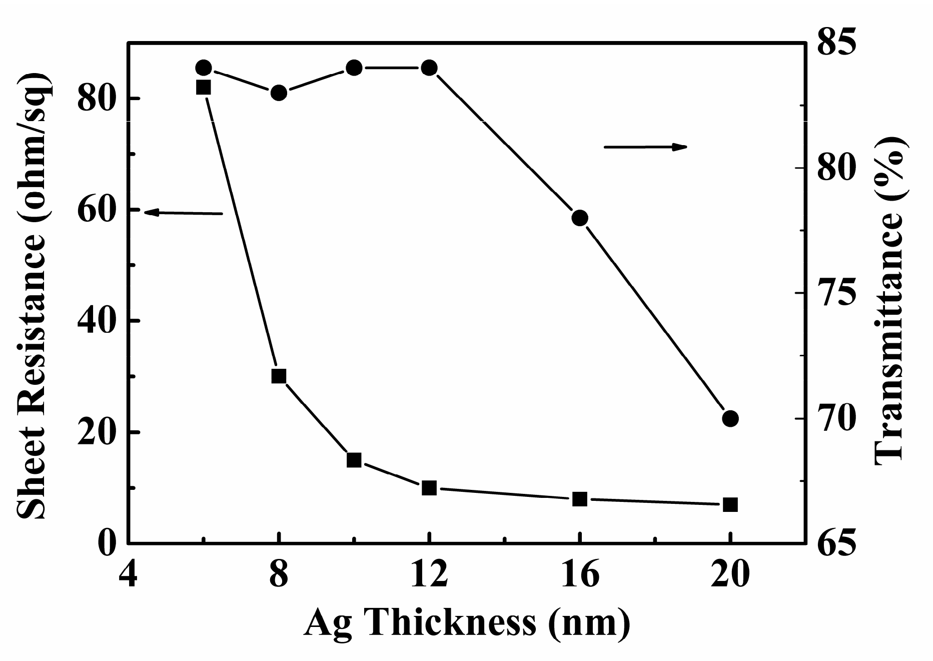

Figure S2. Sheet resistance and maximum transmittance of PAPE electrodes based on glass substrates as a function of Ag thickness.

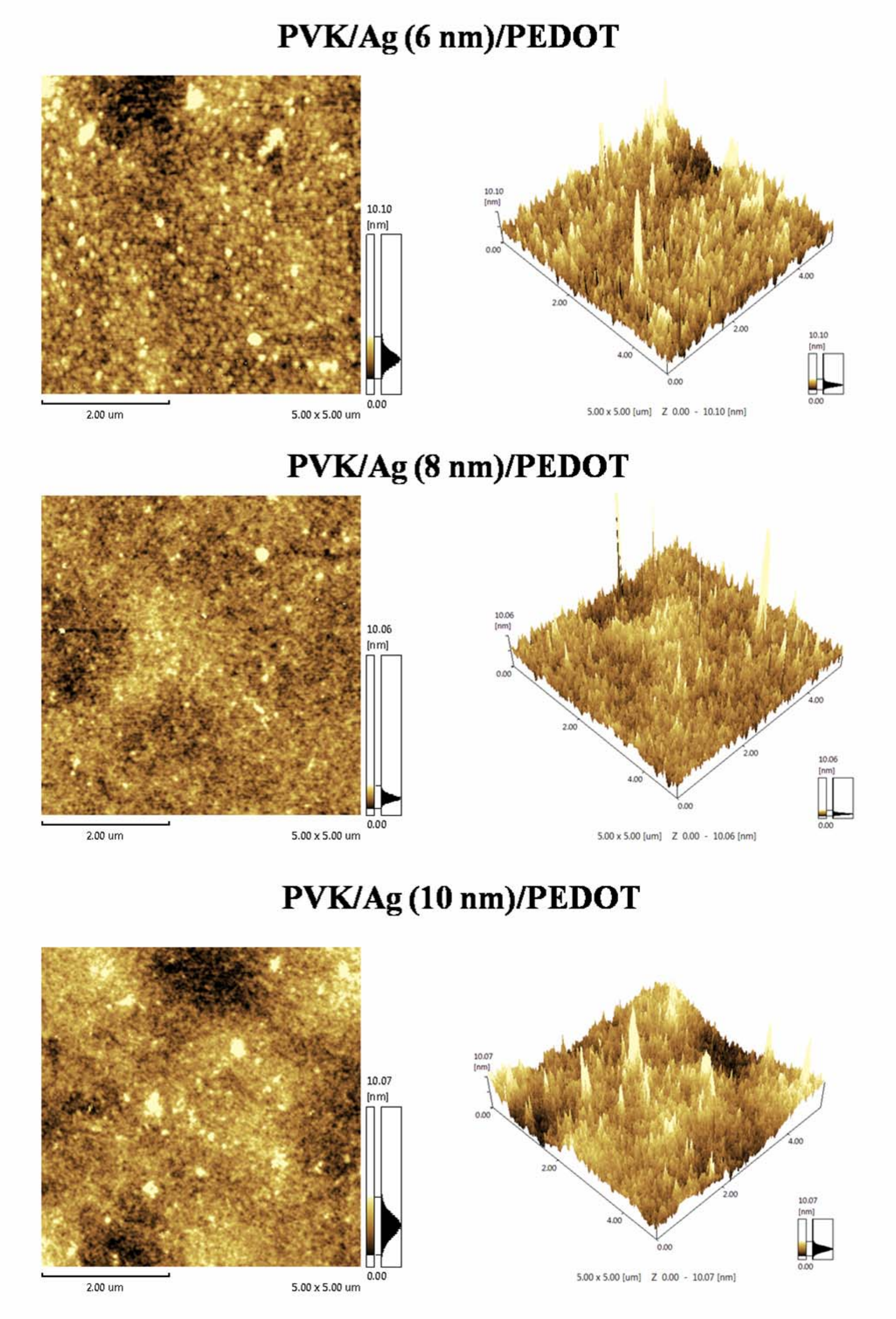

Figure S3. AFM images of PAPE with different Ag thickness.

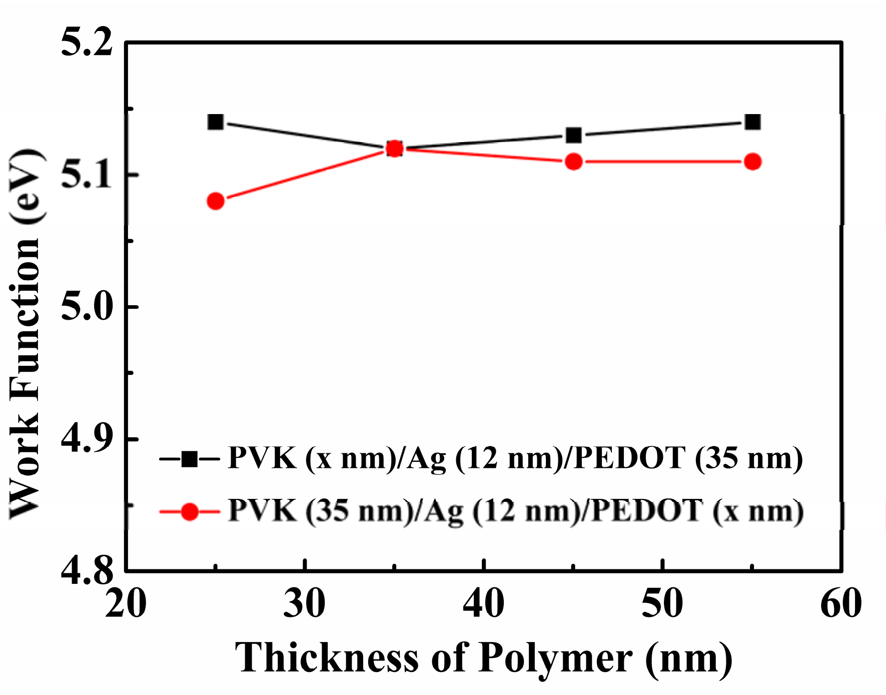

Figure S4. Work function of PAPE electrode as a function of PVK and PEDOT:PSS thickness.

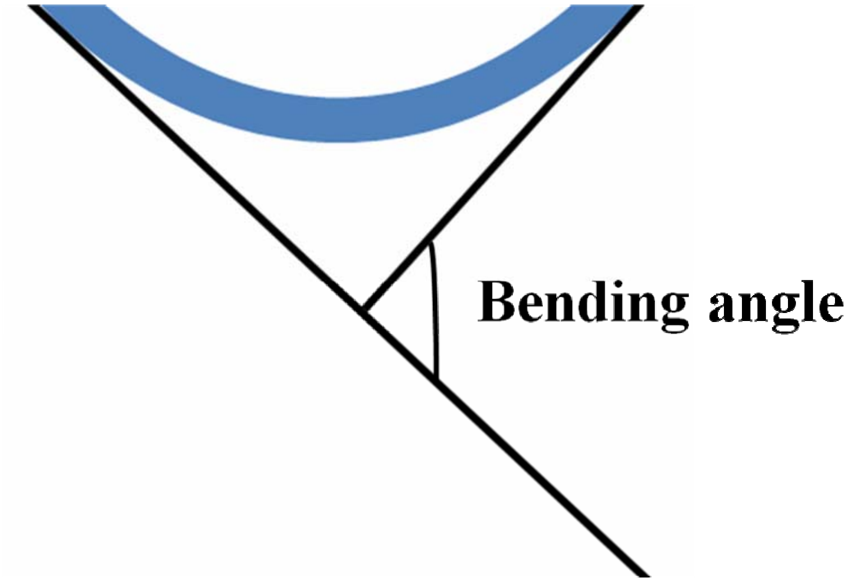

Figure S5. The diagram of bending angle defined in the paper.

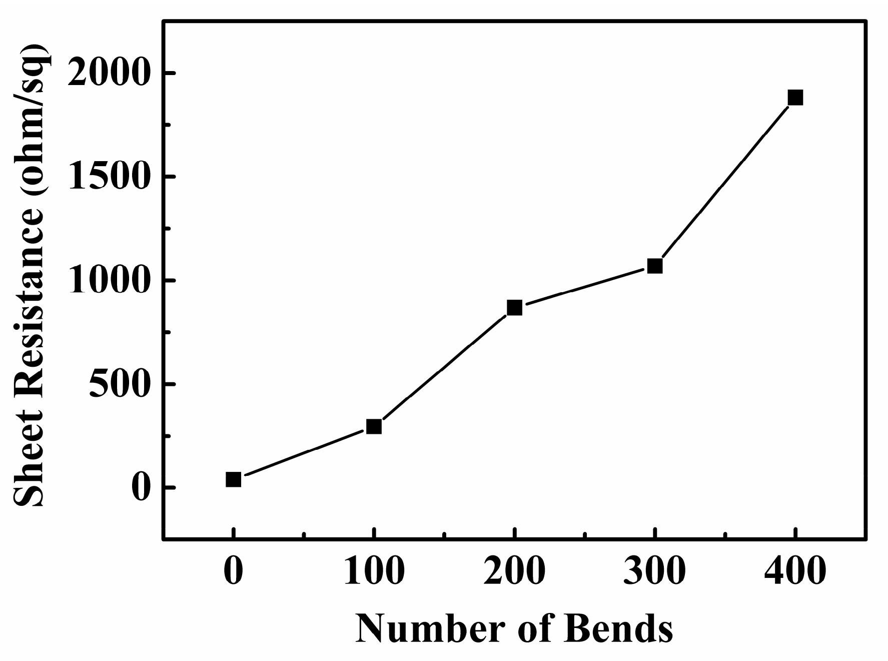

Figure S6. The sheet resistivity of PET/ITO flexible electrode under repeated bending. The bending angle is 90°.

(a)

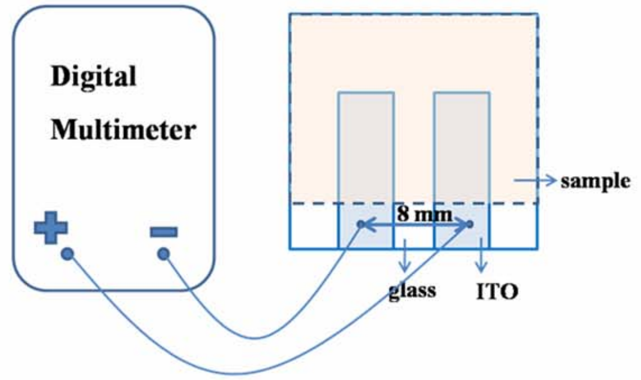

Sample	ITO/PVK	ITO/PEDOT	ITO/Ag	ITO/PVK/Ag	ITO/PVK/Ag /PEDOT
Resistance (Ω)	——	——	36	63	69

(b)

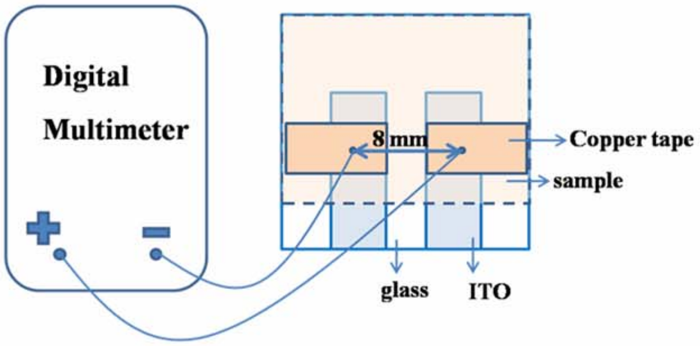

Sample	ITO/PVK	ITO/PEDOT	ITO/Ag	ITO/PVK/Ag	ITO/PVK/Ag /PEDOT
Resistance (Ω)	——	——	33	38	48

(c)

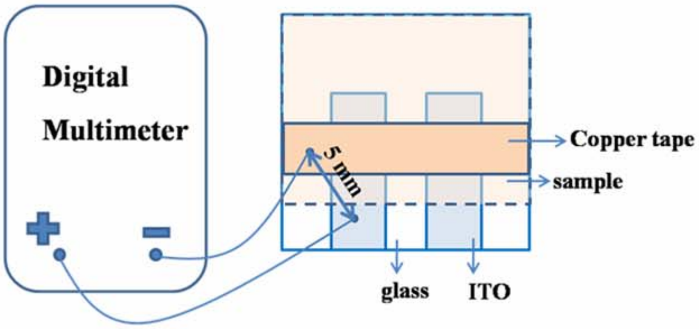

Sample	ITO/PVK	ITO/PEDOT	ITO/Ag	ITO/PVK/Ag	ITO/PVK/Ag /PEDOT
Resistance (Ω)	——	——	25	43	36

Figure S7. Different resistance measurements for different films. Resistance measurements between (a) two ITO films, (b) two pieces of coper tapes and (c) ITO and copper tape.

Film samples (in the region around by the dash) with different structure have been deposited on the pattened ITO glass substrates, and resistance measurements have been carried out with a digital multimeter as shown in Fig. S7. In Figure S7(a), the resistances were measured through two ITO electrodes. For the PVK or PEDOT film, the resistance is over 2000 MΩ between the two bottom separated ITO electrodes, which is too large to be measured by our digital multimeter. But when a Ag layer was deposited on PVK film, or inserted between the PVK and PEDOT, resistance of 63 or 69 Ω was achieved which confirmed that the OMO electrode mainly conducted by the middle metal layer. In Figure S7(b), the resistances were measured through two top separated copper tape electrodes. Similar phenomenon was found as the aforementioned measurements in Fig. S7(a). The both measurements in Fig. S7(a) and (b) indicate that the OMO electrode is conductive either from the top or the bottom organic dielectric layer. In Fig. S7(c), the resistances were measured between the bottom ITO and the top copper tape electrodes, which illustrates that the OMO electrode is conductive between the top and the bottom organic dielectric layers. The above results imply that the OMO electrode is conductive on both of the horizontal and the vertical directions. While, the exact conductive mechanism of the OMO electrode is not yet clear, and need further study.

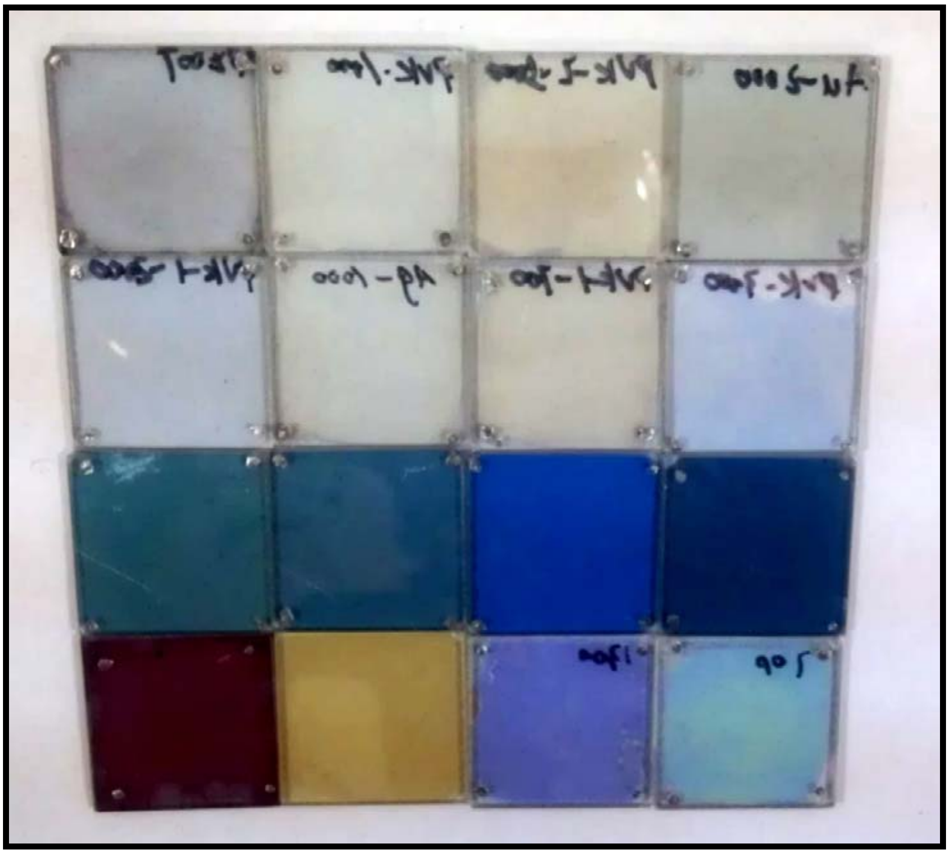

Figure S8. Photographs of OMO multilayer conductive films with different organic and metal materials.

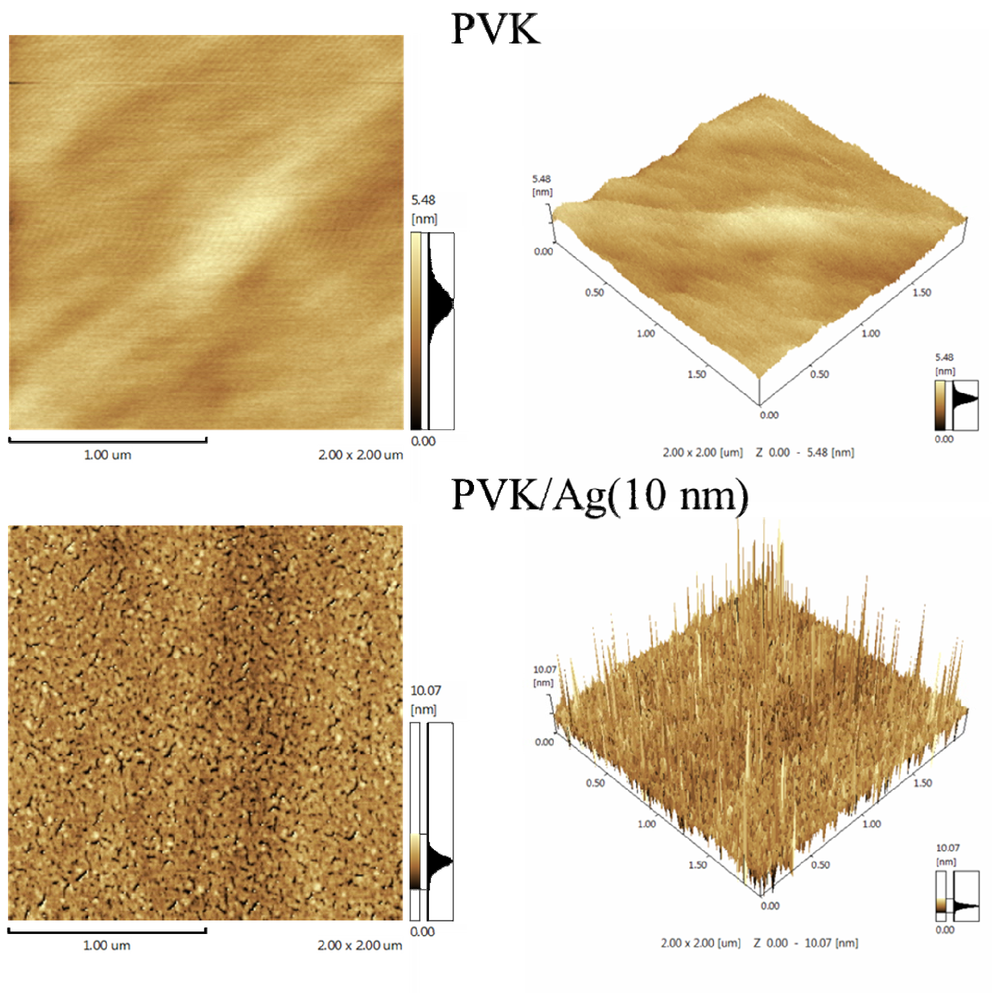

Figure S9. AFM images of PVK and PVK/Ag films on glass substrates.

Table S1. Sheet resistance of glass/PAPE and PET/PAPE film after repeated taping.

Sample \ Taping number	0	1	2	3
Glass/PAPE	10 Ω/sq	10 Ω/sq	10 Ω/sq	10 Ω/sq
PET/PAPE	13 Ω/sq	13 Ω/sq	13 Ω/sq	13 Ω/sq

The AFM images of PVK and PVK/Ag films have been shown in Figure S9. The surface of the PVK film is smooth and the roughness is about 0.5 nm. When a 10 nm Ag was deposited on the PVK, a successive Ag layer can be seen in the AFM figure, and the roughness slightly

increased to 1.9 nm. It illustrates that the smooth PVK film can provide a favorable environment for Ag growth.

The wettability between two films can be reflected through the mechanical adhesion. So the mechanical adhesion of the PAPE film on glass and PET were tested by taping with a tape, and the results have been shown in Table S1. The resistances of PAPE films on glass substrate and PET substrate were all unchanged after taping with a tape for three times. It illustrates that the adhesions between each layer of the PAPE film are large enough to against the tape.

Optical simulation

The characteristic matrix of PAP or PAPE on a glass substrate is [S1]

$$\begin{bmatrix} B \\ C \end{bmatrix} = \left\{ \prod_{j=1}^{3} \begin{bmatrix} \cos\delta_j & \frac{i}{\eta_j}\sin\delta_j \\ i\eta_j \sin\delta_j & \cos\delta_j \end{bmatrix} \right\} \begin{bmatrix} 1 \\ \eta_4 \end{bmatrix} , \qquad \text{(S1)}$$

$$\eta_j = \begin{cases} N_j / \cos\theta_j & \text{For p-polarized wave} \\ N_j \cos\theta_j & \text{For s-polarized wave} \\ N_j & \text{For normal light incidence} \end{cases} \qquad \text{(S2)}$$

where j = 0 (for incidence medium); 1, 2, or 3 for OMO layers; or 4 for the substrate layer. The angular phase thickness is $\delta_j = \frac{2\pi}{\lambda} N_j d_j \cos\theta_j$. θ_j is the angle of wave propagation in the layer as determined from Snell's law. N_j denotes the refractive index of each layer, which is relevant to the incident wavelength λ. The physical thicknesses of the plate PVK (or PEDOT:PSS) exposed to air, the Ag plate, and the PVK plate on the glass substrate are represented by d_1, d_2, and d_3, respectively. The transmittance can be given as

$$T = \frac{4\eta_0\eta_4}{(\eta_0 B + C)^2} \quad , \qquad \text{(S3)}$$

Here, we only consider vertical incidence, thus, $\delta_j = \frac{2\pi}{\lambda} N_j d_j$, and $\eta_j = N_j$. Using data of Ag, PVK and PEDOT:PSS from the literatures,[S2-S4] we can simulate transmittance for PAP or PAPE with glass N_4=1.52 or PI N_4=1.75 as a substrate and under vertical incidence, by inputting j=1, 2, 3, $N_1 = n_1(\lambda)-ik_1(\lambda)$, $N_2 = n_2(\lambda)-ik_2(\lambda)$, $N_3 = n_3(\lambda)-ik_3(\lambda)$, N_4=1.52 (or N_4=1.75) using a computer.

References

S1) Thelen, A. Design of Optical Interference Filters ; McGraw-Hill: New York, 1989.

S2) Lide, D. R. CRC handbook of chemistry and physics ; CRC: Boca Raton, 2008.

S3) Mark, J. E. Polymer data handbook; Oxford University Press: New York,1999.

S4) Mauger, S. A.; Moule, A. J. Characterization of new transparent organic electrode materials, *Org. Electron.* **2011**, *12*, 1948-1956.
